# *In Vivo* Comparison of the Bone Regeneration Capability of Human Bone Marrow Concentrates vs. Platelet-Rich Plasma

**DOI:** 10.1371/journal.pone.0040833

**Published:** 2012-07-12

**Authors:** Weijian Zhong, Yoshinori Sumita, Seigo Ohba, Takako Kawasaki, Kazuhiro Nagai, Guowu Ma, Izumi Asahina

**Affiliations:** 1 Department of Regenerative Oral Surgery, Graduate School of Biomedical Sciences, Nagasaki University, Nagasaki, Japan; 2 Department of Oral and Maxillofacial Surgery, College of Stomatology, Dalian Medical University, Dalian, Liaoning, China; 3 Transfusion and Cell Therapy Unit, Nagasaki University Hospital, Nagasaki, Japan; University of Minho, Portugal

## Abstract

**Background:**

Bone marrow aspirate concentrate (BMAC) including high densities of stem cells and progenitor cells may possess a stronger bone regenerative capability compared with Platelet-rich plasma (PRP), which contains enriched growth factors. The objective of this study was to evaluate the effects of human BMAC and PRP in combination with β-tricalcium phosphate (β-TCP) on promoting initial bone augmentation in an immunodeficient mouse model.

**Methodology/Principal Findings:**

BMAC and PRP were concentrated with an automated blood separator from the bone marrow and peripheral blood aspirates. β-TCP particles were employed as a scaffold to carry cells. After cell counting and FACS characterization, three groups of nude mice (BMAC+TCP, PRP+TCP, and a TCP control) were implanted with graft materials for onlay placement on the cranium. Samples were harvested after 4 weeks, and serial sections were prepared. We observed the new bone on light microscopy and performed histomorphometric analysis. After centrifugation, the concentrations of nucleated cells and platelets in BMAC were increased by factors of 2.8±0.8 and 5.3±2.4, respectively, whereas leucocytes and platelets in PRP were increased by factors of 4.1±1.8 and 4.4±1.9, respectively. The concentrations of CD34-, CD271-, CD90-, CD105-, and CD146-positive cells were markedly increased in both BMAC and PRP. The percentage of new bone in the BMAC group (7.6±3.9%) and the PRP group (7.2±3.8%) were significantly higher than that of TCP group (2.7±1.4%). Significantly more bone cells in the new bone occurred in sites transplanted with BMAC (552±257) and PRP (491±211) compared to TCP alone (187±94). But the difference between the treatment groups was not significant.

**Conclusions/Significance:**

Both human BMACs and PRP may provide therapeutic benefits in bone tissue engineering applications. These fractions possess a similar ability to enhance early-phase bone regeneration.

## Introduction

The regeneration and reconstruction of missing bone in patients with persistent bone defects may be difficult to achieve without interventions such as bone grafting. Different techniques utilizing autologous bone and allografts, xenografts, and various artificial bone substitutes have been developed. However, these techniques have drawbacks and have shown limited success [1]. The need for a more effective regenerative approach led to the development of tissue engineering techniques that usually involve one or more of the following three key elements: scaffold or supporting matrices; growth factors or signaling molecules; and cells [2]. Because only a small amount of tissue from the patient is required, bone reconstruction with this technique is less invasive and safer than conventional methods.

Platelet-rich plasma (PRP) enhances osteogenesis and accelerates healing of an existing wound because growth factors are released from platelets after the coagulation process is locally triggered in the wound site [3]–[5]. The growth factors produced by human platelets include platelet-derived growth factor, insulin-like growth factor, transforming growth factor β, basic fibroblast growth factor, epidermal growth factor, and vascular endothelial growth factor [6], [7]. Hence, the use of PRP may not only improve and facilitate the manipulation of particulate grafts but also increase vascular ingrowth and mitogenic effects on bone-forming cells [8], [9].

The stem cells and progenitor cells derived from bone marrow are the most useful sources of autologous cells for bone tissue regeneration [10]–[14]. Recently, bone marrow aspirate concentrate (BMAC) was suggested to contain an enriched population of mononuclear cells (MNCs) and cytokines and has attracted the attention of clinicians [15]. Utilization of BMAC may bypass the time-consuming and technically difficult process of cell expansion and differentiation, enabling both harvesting and transplanting of BMAC during the same surgical procedure [16]. Furthermore, the platelets in BMAC may provide conditions permitting more rapid and effective bone regeneration by mesenchymal stem cells (MSCs).

Well-controlled comparative studies regarding the bone regenerative capability of these two concentrates isolated from peripheral blood and bone marrow remain scarce, and the results are controversial. A clinical study demonstrated that peripheral blood PRP possesses better potential for alveolar bone augmentation compared with bone marrow-derived cells [17]. Conversely, a recent experimental study claimed that PRP shows no beneficial effects on bone formation and that bone marrow MNCs display significant positive effects on bone regeneration compared to PRP [18].

To explore a feasible approach for facilitating the clinical application of bone tissue engineering techniques, the bone regenerative capabilities of human BMAC and peripheral blood PRP were evaluated with an immunodeficient mouse model using β-tricalcium phosphate (β-TCP) as a scaffold. The bone regeneration effects were evaluated histologically after 4 weeks of healing.

## Results

### Cell Recovery

The concentration of bone marrow nucleated cells increased by a factor of 2.8±0.8 from 19.8±8.2×10^6^/ml to 58.8±33.6×10^6^/ml. White blood cells in peripheral blood increased by a factor of 4.1±1.8 after centrifugation from 4.9±1.5×10^6^/ml to 18.4±5.2×10^6^/ml. Platelets were enriched by a factor of 5.3±2.4 in bone marrow from 12.9±5.2×10^7^/ml to 67.2±37.6×10^7^/ml, and by 4.4±1.9 times in peripheral blood from 18.4±3.8×10^7^/ml to 76.7±25.0×10^7^/ml **(** **Table 1** **)**.

**Table 1:** Cell and platelet concentration before and after centrifugation.

Donor	Cells in BM			Cells in PB			Platelets in BM			Platelets in PB		
	Pre10^6^/ml	Post10^6^/ml	Ratetimes	Pre10^6^/ml	Post10^6^/ml	Ratetimes	Pre10^7^/ml	Post10^7^/ml	Ratetimes	Pre10^7^/ml	Post10^7^/ml	Ratetimes
1	16	30	1.9	4.5	25	5.6	8.4	18	2.1	14.1	105	7.6
2	23	69	2.9	6.3	16	2.5	13.5	70.2	5.2	22.1	76	3.5
3	16	62	3.8	3.1	20	6.5	6.8	57	8.4	15.1	52	3.4
4	11.4	25	2.2	3.8	11	2.9	18.4	123	6.7	18.2	52	2.9
5	32.5	108	3.3	6.6	20	3.0	17.5	68	3.9	22.4	98.5	4.4
Mean ± SD	19.8±8.2	58.8±33.6	2.8±0.8	4.9±1.5	18.4±5.2	4.1±1.8	12.9±5.2	67.2±37.6	5.3±2.4	18.4±3.8	76.7±25.0	4.4±1.9

BM - bone marrow; PB - peripheral blood.

No significant differences were found between the rates of cell concentration increase in bone marrow and peripheral blood (p = 0.25). Although the cell concentrations were variable among donors before and after centrifugation, similar tendencies were exhibited. No significant correlations were found between cell recovery rate and age or gender.

### Cell Characterization

**Figure 1:**
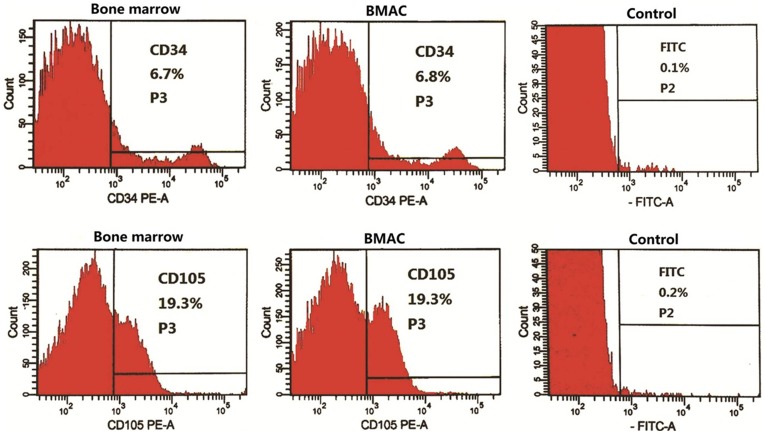
Representative results of FACS analysis. The data of CD34- and CD105-positive cells in bone marrow and the associated isolated fractions came from donor 1. No apparent changes were present in the proportion of CD34- (6.7% to 6.8%) and CD105- (19.3% to 19.3%) positive cells between bone marrow and the BMAC fractions post-isolation. The proportions of FITC-positive cells in the isotype controls were 0.1% and 0.2%, respectively.

FACS analysis showed that the cell populations from bone marrow and peripheral blood were both positive for well-known stem cell markers including CD34, CD90, CD105, CD271, and CD146 **(** **Figure 1** **)**. The proportion of FITC-positive cells in the isotype control was less than 0.3%. Over 92% of bone marrow cells and isolated cell products were positive for CD45. After centrifugation, the percentage of the different stem cells varied in relation to donor and cell categories, possibly reflecting the heterogeneity of the original population **(Table S1)**.

Generally, the proportions of CD marker positive cell changed slightly after centrifugation according to the averaged FACS values **(** **Table 2** **)**. No significant statistical differences were found between CD marker positive cell proportion before and after centrifugation.

**Table 2:** Averaged data of FACS results (mean±SD).

	CD34 (%)	CD271 (%)	CD90 (%)	CD105 (%)	CD146 (%)
BM	6.8±6.2	1±0.6	5.8±4.9	19.2±16.8	3.4±3.9
BMAC	7.2±6.9	0.6±0.3	7.5±7.2	18.9±16.1	3.9±2.5
PB	0.48±0.6	0.2±0.2	3.1±3.1	4.6±6.3	1.4±1.1
PRP	2.1±2.2	0.6±0.8	3.1±3.2	5.5±6.8	5.3±9.6

### Histological Analysis

**Figure 2:**
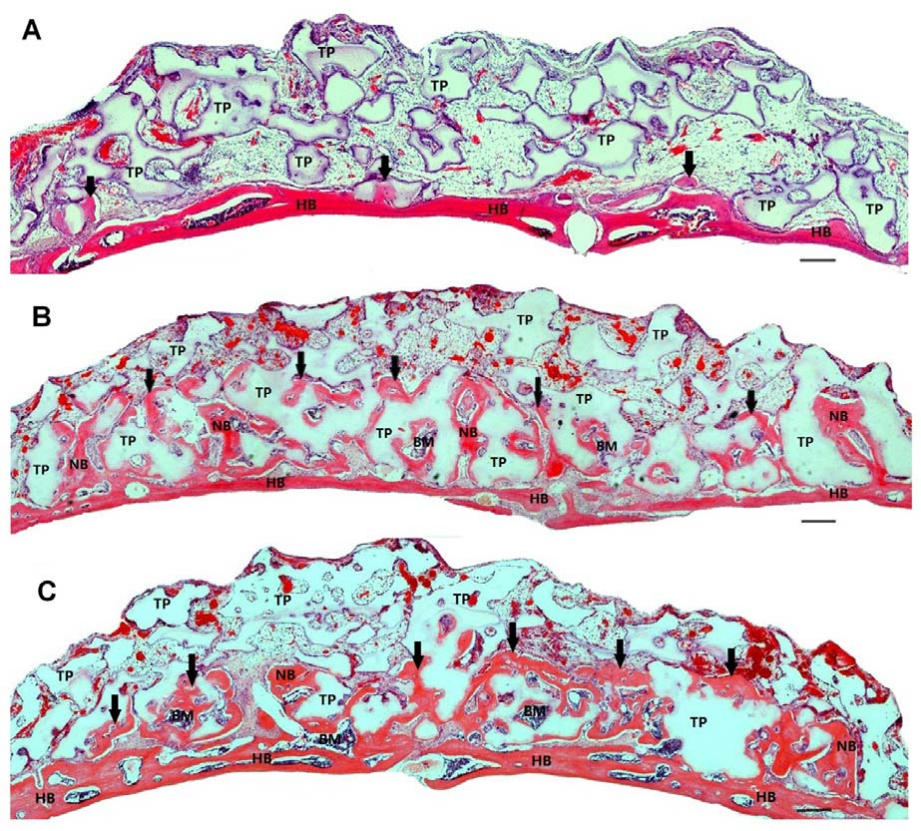
Typical histological micrographs of newly formed bone 4 weeks after transplantation. Coronal plane sections were stained with hematoxylin and eosin. (A): β-TCP alone induced only a limited amount of new bone formation, and most particles were embedded in loose connective tissue. (B) and (C): A large amount of new bone and immature bone marrow were generated on the calvarial bone of mice transplanted with BMAC (B) or PRP (C). The newly formed bone was sufficiently integrated with the host bone. The TCP particles were in the process of being resorbed and replaced with newly formed bone. The scale bars represent 100 µm. Abbreviations: HB, host bone; NB, new bone, also indicated by arrows; BM, bone marrow; TP, β-TCP particle.

**Figure 3:**
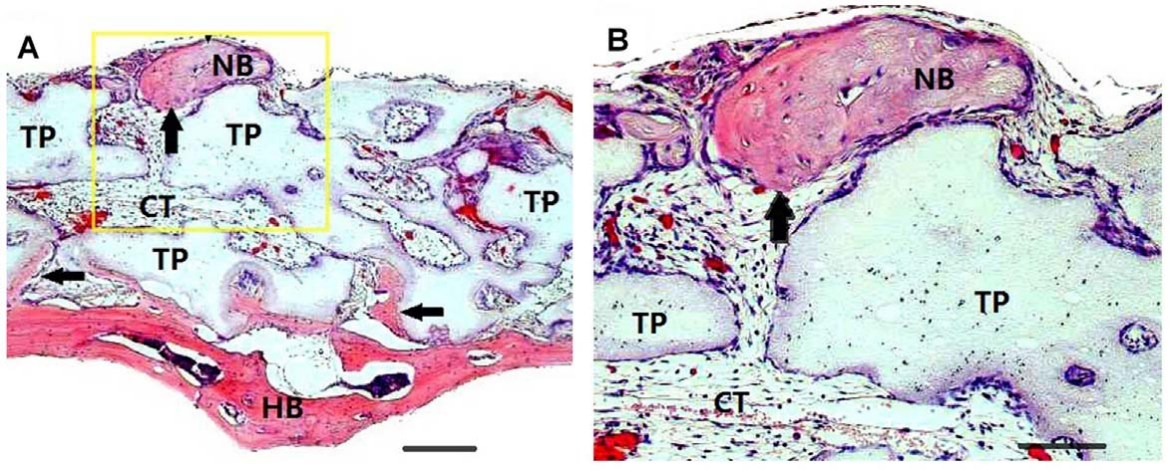
Hematoxylin and eosin staining shows new bone formation induced by BMAC. (A): Isolated bone was formed at the peripheral area of the transplant material and far from the calvarial bone. (B): magnification of the outlined area (A). Scale bars in (A) represent 100 µm; scale bars in (B) represent 50 µm. Abbreviations: HB, host bone; NB, new bone, also indicated by arrows; TP, β-TCP particle; CT, connective tissue.

Four weeks after implantation, a small amount of new bone was found in the immediate proximity of the host bone in the TCP control group **(** **Figure 2A** **)**. However, the augmented area was mostly filled with β-TCP particles, which were embedded in loose connective tissue, without bone formation. In the PRP and BMAC groups, considerably more new bone was present in areas close to the host bone, and the new bone surrounded the β-TCP particles and connected with the host bone **(** **Figure 2B, 2C** **)**. Newly formed bone was also observed in the macropores of the β-TCP particles. Blood vessels could be observed throughout the specimens, showing that the blood supply was abundant. Moreover, isolated new bone islands far from the host bone were observed in the serial sections of some samples transplanted with BMACs and bone cells in the new bone could be identified on the magnified micrographs **(** **Figures 3A, 3B** **).**

### Histomorphometic Analysis

**Figure 4:**
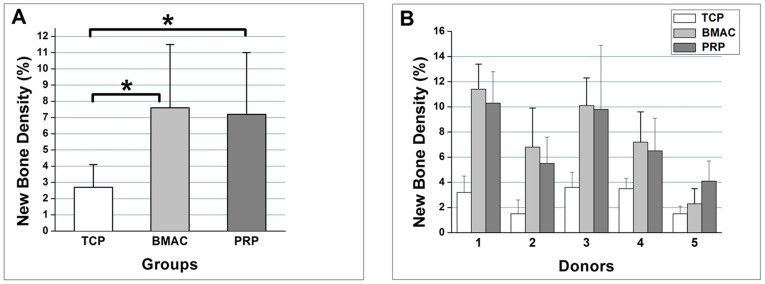
Differences in the percentage of new bone were determined by histomorphometric analysis. (A): Comparison of the total mean value of the percentage of new bone among the β-TCP alone, BMAC, and PRP groups (B): The new bone densities were compared among donors. Donor 5 is female. Significant differences were found between individuals. * indicates a significant difference (p<0.05) between paired groups. Values are the means ± standard deviation from five sections of each sample.

Histomorphometric analysis showed that the average percentage of newly formed bone was 7.6±3.9% in the BMAC group, 7.2±3.8% in the PRP group, and 2.7±1.4% in the TCP group **(** **Figure 4A** **)**. BMACs formed slightly more new bone than PRP, but these differences were not statistically significant. However, the new bone volumes generated in the BMAC and PRP groups were significantly greater than that in the TCP control group (p<0.01). The results of the histomorphometric analysis were confirmed with the counting bone cells in and around the newly formed bone. The bone cell number in the new bone was 552±257 in the BMAC group, 491±211 in the PRP group, and 187±94 in the TCP group **(** **Table 3** **)**. Statistical analysis revealed that the numbers of bone cells in the BMAC and PRP group were significantly higher than that in the TCP control group (p<0.01). No significant differences were found between the bone cell numbers in the BMAC and PRP group though slightly more bone cells were observed in the new bone of BMAC group.

In both treatment groups, the new bone percentages and the bone cell number in the new bone, changed in the same tendency and were twice as high as that in the TCP group. Significant differences were observed in new bone formation among the donors for each treatment group (p<0.05). Specifically, the PRP from the female donor produced more bone cells than the BMAC, but the yields of new bone by aspirate from the female donor were slightly lower than aspirate from the male donors **(** **Figure 4B** **)**.

## Discussion

The application of uncultured bone marrow-derived cells to improve tissue regeneration may avoid the risks associated with in vitro expansion of stem cells. Previous studies in bone and cartilage tissue engineering have shown significant therapeutic benefits associated with the use of uncultured marrow-derived cells [19]–[23]. Aside from MSCs, human bone marrow MNCs contain sub-populations of various types of progenitor cells, including endothelial progenitor cells, which are thought to play an important role in angiogenesis [24]. These two factors may contribute to the promotion of bone regeneration [25]. Recently, BMAC, which contains concentrated MNCs and platelets, was proposed as a new “platinum standard” for bone reconstruction [26].

In the present study, the harvesting process and concentrating procedure for BMAC and PRP were performed entirely under a formal clinical setting, and the bone graft substitute we used, β-TCP, is a commercially available product approved for human use. All these factors are relevant to further clinical application. Both isolated portions contained highly concentrated MNCs and platelets suspended in plasma. The results of cell counting verified that sufficient numbers of such cells were obtained by this separation machine compared to values reported in other studies [27]. FACS analysis confirmed the effectiveness of this isolation method. Although the proportions of CD34-, CD217-, CD90-, CD105-, and CD146-positive cells were not significantly increased in the centrifuged fractions, it is plausible to speculate that the numbers of CD marker positive cell were actually increased after centrifugation since total cell concentrations had been enriched by 2.8±0.8 and 4.1±1.8 times in the fraction of BMAC and PRP.

The small amount of new bone formation in the TCP group was probably secondary to osteoconduction, which involves the migration of mouse osteogenic progenitor cells from the calvarial bone into the TCP scaffold [28]. Bone formation generated through this process is relatively slow, and as expected, much less bone was formed in the TCP transplants compared with BMAC and PRP transplants. The amount of newly formed bone at 4 weeks in the BMAC group was 7.6±3.9%, was significantly enhanced compared to the TCP control group (2.7±1.4%). This result is consistent with previous animal experiment results [25], [29], which showed that transplantation of isolated bone marrow MNCs induced greater bone formation with new bone percentage from 18.4% to 27.5% at 8 weeks, although the MNCs used were derived from rabbit and bone defects were made on the femoral condyle with atelocollagen gel as scaffold. In the field of maxillofacial bone grafting, bone marrow derived MNCs with or without concentrating have been used for sinus elevation and alveolar bone augmentation and produced a significant viable bone [30], [31]. It was reported that BMAC combined with bovine bone mineral achieved 12.6±1.7% new bone during sinus augmentation after 3–4 months of healing [16]. In our experiment, short healing time of 4 weeks and onlay transplantation with less blood supply may explain the results of relatively lower bone formation involving human cells.

Since the growth factors released from platelets are capable of promoting cell proliferation and angiogenesis, PRP from peripheral blood has been widely used in pre-clinical and clinical applications to enhance bone regeneration, but the results have been inconsistent [32]–[38]. Variation in PRP preparation protocols may explain the controversy. The standard protocol for PRP preparation is as follows: moderate platelet concentration 4- to 8-fold above the physiological level, PRP activation with the proper dosages of bovine thrombin and calcium chloride, and use of a suitable bone filler as a carrier for growth factors released from the platelets [39]. The PRP preparation in our experiment basically conformed to this standard protocol, and the percentage of newly formed bone in the PRP group (7.2±3.8%) was significantly greater than that of the TCP group (2.7±1.4%).

Comparative studies of the capability of BMAC and PRP in bone regeneration are scarce. One clinical study showed that PRP is more effective than bone marrow MNCs in alveolar bone augmentation [17]. However, a recent experimental study indicated that uncultured bone marrow MNCs displayed significant positive overall effects on bone regeneration compared to PRP [18].

Our data demonstrated that BMAC composed of enriched nucleated cells and platelets generated a slightly more bone cells in the new bone than PRP, but the differences were not significant. Moreover, newly formed bone was mainly present in areas close to the host bone due to osteoconduction. Isolated new bone islands far from the host bone were discovered in only a few samples transplanted with BMAC and were probably produced by the implanted cells through osteoinduction. In addition, the data presented here did not indicate a correlation between the number of cells obtained from each fraction and the amount of bone formation. Actually, the number of MNCs in BMAC was three times as much as that in PRP, but the number of platelets in both groups was similar. Therefore, we speculate that the number of MSCs was not the most important factor in the process of bone regeneration. Instead, it is possible that platelets and/or fibrin play a significant role in this model, and the dominant mechanism may be biosynthetic effects between the transplanted cells and cytokines that chemotactically recruited host osteogenic progenitor cells. The underlying mechanisms require further investigation.

In conclusion, our findings suggest that BMAC and peripheral blood PRP possess similar potential regarding acceleration of new bone formation and increase new bone volume in the early phase of bone regeneration. Therefore, BMAC or PRP combined with β-TCP may be an effective approach for promoting initial bone formation. However, in a regular clinical setting, the transplantation of PRP may be a more feasible method for enhancing bone regeneration. Further studies are being carried out to evaluate the clinical performance of these two fractions.

**Table 3:** Number of bone cells in newly formed bone among groups and donors.

Donor	1	2	3	4	5	Mean ± SD
TCP	163±69	107±43	265±91	279±76	122±30	187±94
BMAC+TCP	785±246	522±258	657±255	605±264	190±48	552±257
PRP+TCP	712±235	445±180	524±105	480±171	293±111	491±211

## Materials and Methods

### Ethics Statement and Donor Data

Sample collection was approved by the Ethics Committee of Nagasaki University Graduate School of Biomedical Sciences (Approval No. 11032828), and written informed consent was obtained from all donors. Volunteer donors comprised four males and one female aged 26 to 54 years with no history or evidence of genetic disease or malignancy.

### BMAC and PRP Isolation

Bone marrow was aspirated under local anesthesia from the posterior iliac crest. Three punctures were made to obtain sufficient bone marrow. Peripheral blood was obtained from the cubital vein. Equal volumes (27 ml) of bone marrow and peripheral blood aspirates were collected with syringes containing 4 ml of the anticoagulant citrate dextrose. From each sample, 1 ml was reserved for cell analysis. Then, 3 ml plasma containing concentrated MNCs and platelets was isolated from 30 ml aspirate mixture using an automated blood cell separator (Magellan MDK 305, Ateriocyte Medical System Inc., Cleveland, OH, USA) according to the manufacturer instructions. A small fraction of the centrifuged cell suspension was reserved for cell analysis.

### Cell Characterization

Before and after centrifugation, cell analysis was conducted by cell counting and fluorescence activated cell sorting (FACS). Cell counting was performed using an automatic blood counter (Sysmex XE2100, Sysmex Co., Kobe, Japan). MSC phenotypes were detected with flow cytometry (BD FACS Canto II flow cytometer, Becton Dickinson, Franklin Lakes, NJ, USA). The monoclonal antibodies CD34, CD271, CD90, CD105, CD146, and CD45-FITC (Becton Dickinson Monoclonal Center Inc., Mountain View, CA, USA) conjugated to fluorescent molecules, were employed.

### Animal Experiment

All animal experiments were performed at the Animal Center of Nagasaki University, and Guidelines for Animal Experimentation were observed. All procedures were approved by Biomedical Research Center (BRC) of Nagasaki University (Approval No. 080140706). Surgery was performed on 60 healthy, 6-week-old female BALB/cAJcl-nu/nu mice (Nihoncrea, Tokyo, Japan) divided into three groups: BMAC+β-TCP, PBPRP+β-TCP, and β-TCP (control). Isolates from each donor were administered to four mice in each group.

β-TCP granules (Osferion®; Olympus, Tokyo, Japan) of size 0.5 mm−1.5 mm were used as cell carriers. Each portion of bone graft material comprised 20 mg β-TCP and 100 µl cell concentrate containing approximately 5.0×10^6^ MNCs. Before transplantation, 10 µl bovine thrombin and 10% calcium chloride mixture (1∶1 ratio) was added to the β-TCP/cell mixture to trigger fibrin polymerization to produce an insoluble gel. The final concentrations of thrombin and CaCl_2_ in the grafting aspirates were 227.3 U/ml and 4.6 mg/ml, respectively. β-TCP mixed with physiological saline was used as a negative control.

Under inhalation anesthesia with diethyl ether, the calvarial skin was incised, and the periosteum reflected. After transplanting the graft materials onto the cranium surface, the incision was sutured. Four weeks after surgery, the animals were sacrificed by CO2 asphyxiation and the transplants were harvested.

### Histomorphometrical Analysis of the Transplants

The harvested samples were fixed in 4% neutral formaldehyde followed by decalcification in disodium EDTA and embedding in paraffin. Serial 5-µm coronal sections were prepared every 100 µm and stained with hematoxylin and eosin. Five sections through the center of the transplants were selected from each sample for morphometric assessment. The host bone is dark pink and the new bone is light pink. The volume of newly formed bone was analyzed with Scion Image software (NIH, Bethesda, MD, USA) and the number of bone cells within in the new bone, which seems to be osteocytes, was counted. **(Figure S1)** Bone regeneration at the implanted sites was evaluated by the quantification of bone cells and the density of new bone, which was expressed as the percentage of new bone area to the total implant area.

### Statistical Analysis

Results were recorded as mean values ± standard deviation. Statistical analysis of differences between groups was performed using one-way ANOVA with SPSS 11.0 software (SPSS Inc., Chicago, IL, USA). Probability (p) values less than 0.05 were considered significant.

## Supporting Information

Figure S1 **Method for Counting bone cells.** (A): To identify the new bone areas and separate them from the host skull bone. The new bone could be judged by slightly lighter color and less mature structure compared to the host bone. (B): To outline and isolate the new bone areas with the help of the graphics software (Photoshop). (C): To mark the bone cells with red spots. The bone cells included in the evaluation were those which were embedded in the new bone area.
(TIF)

Table S1 Combined data of FACS results of all donors.
(DOCX)
