# Supplementary figures and images for: In Vivo Comparison of the Bone Regeneration Capability of Human Bone Marrow Concentrates vs. Platelet-Rich Plasma
Source: PLoS One. 2012 Jul 12;7(7):e40833. doi: 10.1371/journal.pone.0040833 (PMC3395629; doi:10.1371/journal.pone.0040833)

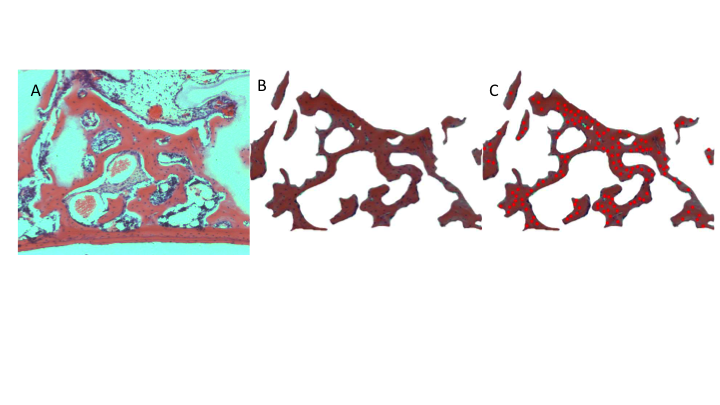

Supplement: Figure S1 — Method for Counting bone cells. (A): To identify the new bone areas and separate them from the host skull bone. The new bone could be judged by slightly lighter color and less mature structure compared to the host bone. (B): To outline and isolate the new bone areas with the help of the graphics software (Photoshop). (C): To mark the bone cells with red spots. The bone cells included in the evaluation were those which were embedded in the new bone area. (TIF) [file pone.0040833.s001.tif]
