# Supplementary material for: In Vivo Comparison of the Bone Regeneration Capability of Human Bone Marrow Concentrates vs. Platelet-Rich Plasma
Source: PLoS One. 2012 Jul 12;7(7):e40833. doi: 10.1371/journal.pone.0040833 (PMC3395629; doi:10.1371/journal.pone.0040833)
Supplement: Table S1 — Combined data of FACS results of all donors. (DOCX) [file pone.0040833.s002.docx]

**Supplemental Table 1** Combined data of FACS results of all donors. (supplemental data)

| **Donor** | **CD34 (%)** | | | | **CD271 (%)** | | | | **CD90 (%)** | | | | **CD105 (%)** | | | | **CD146 (%)** | | | |
| --- | --- | --- | --- | --- | --- | --- | --- | --- | --- | --- | --- | --- | --- | --- | --- | --- | --- | --- | --- | --- |
|  | **BM** | **BMAC** | **PB** | **PRP** | **BM** | **BMAC** | **PB** | **PRP** | **BM** | **BMAC** | **PB** | **PRP** | **BM** | **BMAC** | **PB** | **PRP** | **BM** | **BMAC** | **PB** | **PRP** |
| **1** | **6.7** | **6.8** | **1.1** | **5.4** | **1.1** | **1.1** | **0.2** | **0.5** | **8.7** | **1.1** | **3.7** | **0.5** | **19.3** | **19.3** | **13.8** | **16** | **1.3** | **1.3** | **0.8** | **1** |
| **2** | **17.1** | **18.8** | **0** | **0.5** | **1.7** | **0.7** | **0.4** | **2** | **2.1** | **6.5** | **0.6** | **2** | **44.7** | **46.4** | **0** | **0.5** | **10.1** | **7.5** | **2.7** | **22.4** |
| **3** | **4.3** | **5** | **0.1** | **1.1** | **0.3** | **0.4** | **0** | **0.1** | **1** | **0.7** | **0.4** | **0.4** | **7.8** | **7.8** | **0.1** | **0.9** | **3.3** | **2.9** | **0.9** | **1.3** |
| **4** | **0.6** | **0.5** | **0** | **0.2** | **0.4** | **0.5** | **0.1** | **0.2** | **4.6** | **11.5** | **2.8** | **5** | **0.8** | **7.4** | **0.4** | **1.5** | **0.2** | **5.3** | **0.1** | **0.4** |
| **5** | **5.1** | **4.7** | **1.2** | **3.3** | **1.4** | **0.5** | **0.5** | **0.6** | **12.8** | **17.6** | **8.1** | **7.7** | **23.3** | **13.5** | **8.7** | **8.7** | **1.9** | **2.3** | **2.5** | **1.5** |
| **Mean**  **±**  **SD** | **6.8**  **±**  **6.2** | **7.2**  **±**  **6.9** | **0.48±0.6** | **2.1±2.2** | **1**  **±0.6** | **0.6**  **±0.3** | **0.2±0.2** | **0.6±0.8** | **5.8±4.9** | **7.5**  **±7.2** | **3.1±3.1** | **3.1±3.2** | **19.2±16.8** | **18.9±16.1** | **4.6±6.3** | **5.5±6.8** | **3.4±3.9** | **3.9**  **±2.5** | **1.4±1.1** | **5.3±9.6** |
| **BM-bone marrow; BMAC-bone marrow aspirate concentrate; PB-peripheral blood; PRP-platelet rich plasma** | | | | | | | | | | | | | | | | | | | | |
